# Morphogene adsorption as a Turing instability regulator: Theoretical analysis and possible applications in multicellular embryonic systems

**DOI:** 10.1371/journal.pone.0171212

**Published:** 2017-02-07

**Authors:** Alexey M. Nesterenko, Maxim B. Kuznetsov, Daria D. Korotkova, Andrey G. Zaraisky

**Affiliations:** 1 Shemyakin-Ovchinnikov Institute of Bioorganic Chemistry, Russian Academy of Sciences, Moscow, Russia; 2 Belozersky Institute of Physico-Chemical Biology, Lomonosov Moscow State University, Moscow, Russia; 3 Lebedev Physcal Institute, Russian Academy of Sciences, Leninsky prospect, Moscow, Russia; 4 Department of Embryology, Faculty of Biology, Lomonosov Moscow State University, Moscow, Russia; Bioinformatics Institute, SINGAPORE

## Abstract

The Turing instability in the reaction-diffusion system is a widely recognized mechanism of the morphogen gradient self-organization during the embryonic development. One of the essential conditions for such self-organization is sharp difference in the diffusion rates of the reacting substances (morphogens). In classical models this condition is satisfied only for significantly different values of diffusion coefficients which cannot hold for morphogens of similar molecular size. One of the most realistic explanations of the difference in diffusion rate is the difference between adsorption of morphogens to the extracellular matrix (ECM). Basing on this assumption we develop a novel mathematical model and demonstrate its effectiveness in describing several well-known examples of biological patterning. Our model consisting of three reaction-diffusion equations has the Turing-type instability and includes two elements with equal diffusivity and immobile binding sites as the third reaction substance. The model is an extension of the classical Gierer-Meinhardt two-components model and can be reduced to it under certain conditions. Incorporation of ECM in the model system allows us to validate the model for available experimental parameters. According to our model introduction of binding sites gradient, which is frequently observed in embryonic tissues, allows one to generate more types of different spatial patterns than can be obtained with two-components models. Thus, besides providing an essential condition for the Turing instability for the system of morphogen with close values of the diffusion coefficients, the morphogen adsorption on ECM may be important as a factor that increases the variability of self-organizing structures.

## Introduction

Nonequilibrium (dissipative) or dynamic self-organization is supposed to play a central role in the embryonic patterning [1–3]. Such self-organization leads to the formation of large-scale dynamic structures of different nature that regulates cell differentiation within the developing embryo [4]. The most generally accepted idea is that special secreted proteins, the morphogens, play critical role in the establishment of these spatial structures. In the simplest case, the concentration gradients of morphogens organize patterning of the embryo in the way that different threshold concentrations of a given morphogen switch on different sets of genes [5–7]. As a result, a specific spatial pattern of different cell differentiation types is formed along the morphogen gradient [6].

Self-organizing processes can be described by discrete models based on cellular automata approach [8] or by continuous models based on reaction-diffusion partial differential equations (PDE) approach. The latter can describe self-organisation by PDEs that have spatially non-homogenous solitions. When these solutions are formed spontaneously and remain temporally stable, one says that PDE has ‘Turing instability’. Regardless of specific mechanism, two conditions are critical for self-organization of the large-scale spatial structures in the initially homogeneous system [9]. First, there should be nonlinear relationships between substances responsible for the formation of the pattern. Second, the system must involve at least two agents and one of them must diffuse slower than the other. The most simple models, which demonstrate Turing instability, consist of two reaction-diffusion differential equations and describe the formation of stable gradients of two hypothetical substances called “activator” and “inhibitor”. These substances have nonlinear interactions with each other and diffuse with sharply different rates: the activator slowly and the inhibitor fast. One of the most well-known models of this kind, which was proposed to describe the formation of stable gradients in biological objects, is the Gierer and Meinhardt model (GM) [7, 10]. The first necessary condition for the Turing-type self-organization, namely the nonlinear interaction between the inhibitor and the activator, holds due to the nonlinear response of the gene network encoding the proteins that play roles of the inhibitor and the activator [11, 12]. However, the second condition, i.e. a sharp difference in the diffusion rates, seems to be difficult to achieve unless diffusing protein morphogens have great differences in size. Meanwhile, most of the known morphogens have approximately the same size around 20–30 kDa and thus must demonstrate quite similar rates of free diffusion. Hence, the question of how a sharp difference in the diffusion rates between the activator and the inhibitor could be achieved in real embryo remains open.

Besides the protein size, a significant factor that may influence the morphogens’ diffusion within the multicellular embryo is the morphogen’s interaction with the components of the extracellular matrix (ECM). In particular, a retardation of the diffusion can result from the adsorption of morphogens on negatively charged ECM components, such as heparan sulfate proteoglycans (HSPG) [13]. The influence of HSPG on the morphogens’ activity was described previously [14, 15]. In support of this, we have shown recently that the interaction of secreted morphogens with HSPG in the intercellular space (IS) of the *Xenopus laevis* embryos can significantly retard the diffusion [16, 17]. As a result, depending on the morphogens’ affinity to ECM, there may appear greater than an order of magnitude difference in the effective diffusion rate between different morphogens within IS [16]. To our knowledge, there are no published works, in which the adsorption of morphogens on HSPG is taken into account when modeling the process of the embryonic self-organization. In the present work we demonstrate how the adsorption could be incorporated to the simple two-component model.

We develop a mathematical model of morphogenesis consisting of three reaction-diffusion equations. Taking as the basis the classical Gierer-Meinhardt model we extend it introducing interaction of activator with ECM. Such modification allows us to obtain stable dissipative structures for morphogens with similar diffusion coefficients. We demonstrate how to apply our modified model to well known Wnt-DKK hair follicle patterning system identifying additional physically relevant parameters. Furthermore, in the spectrum of possible patterns provided in our model appears to be much wider than that of classical two-equation models. For instance, spots that change size gradually can be easily simulated by this model. In particular, we demonstrate how our model can describe pear-like shape of the neural anlage on the base of known adsorption gradient in *Xenopus* gastrula.

Finally, theoretical analysis of the model allows us to determined conditions, under which our extended model can be reduced to only two equations, equivalent to those used in the classical GM model.

## Analysis and simulation of reaction-diffusion models

### What can we figure out through linear analysis of the Turing-type models?

The reaction-diffusion system of equations can be qualitatively studied using “linear analysis” (or “eigenvalue analysis”), that can show whether the Turing-type instability is possible for the given parameter values and provide important information on the behavior of Turing-type systems. However, linear analysis is often omitted in recent studies (e.g. [18, 19]). In the current work we perform linear analysis of both classical and extended GM models in the corresponding parametric spaces.

**Fig 1:**
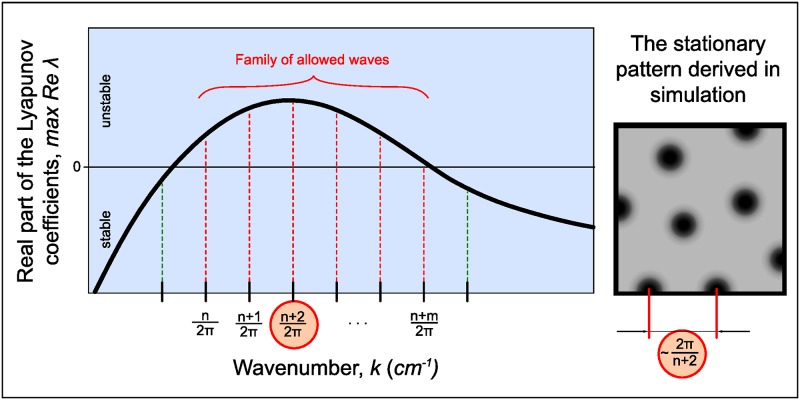
Common shape of the dispersion curve. In the stationary state spatial structures (right pane) have a period which in many cases can be approximately predicted from the dispersion curve (left pane). Self-exited and damping spatial waves are shown with red and dashed lines, respectively.

To understand the idea of the method let us consider a reaction-diffusion system, that models dynamic of concentration of some substance *c*(*x*, *t*). Let us also suppose that corresponding kinetics equations possess a stable stationary point. Then, near the corresponding homogeneous steady state (HSS) evolution of the considered concentration is given by the expression:

$$c\left(x,t\right)=\sum_{k=0}^{\infty }C_{k}\cos\left(kx\right)e^{\lambda (k)t},$$

where *k* are wave numbers and *λ*(*k*) are Lyapunov coefficients that reflect temporal instability of the corresponding wave near HSS. It means that in the complicated space pattern, every wave evolves independently and its growth rate is controlled by parameter *λ*, which is different for different wave numbers. If the stationary pattern is formed as a result of system evolution then there exists the range of wave numbers *k*_*i*_1__,…,*k*_*i*_*n*__ such that Re *λ*(*k*_*i*_*j*__) > 0 for *j* = 1,…,*n*. Thus, function {max **Re** *λ*(*k*)} called dispersion curve gives information about existence of nonuniform stationary patterns and predicts their possible periodicity. The main concept of the linear analysis of Turing-type systems is summarized in Fig 1. The spectrum of instable wave numbers is quantized for the reaction volume considered limited. As the homogenous state should be stable without diffusion, i.e. *Re λ*(0) < 0, the curve at the figure starts from the negative half-space. At the right panel of the figure we drew round spots as an example of the shape of resulting structures, which can not be determined by linear analysis and depends on many factors including space dimensionality. On the contrary, the possible period of initial unstable wavelengths does not depend on reactor dimensionality and can be precisely determined by 1D linear analysis.

In practice the situation is more complicated, as linear analysis has some restrictions. Firstly, to be rigorous we should write Turing conditions for several *λ* (number of *λ* equals number of equations in the system), so that in the range of instable wavenumbers one *λ* should have positive real part and another—negative. Importantly, Turing-type instability (stationary inhomogenity) should be distinguished from wave-type instability (fluctuating inhomogenity), that occurs in three-component system when two *λ*’s real parts become positive [20], and from combinations of these instability-types, possible in complex systems [21]. Since these scenarios are rarely used for modeling biological morphogenesis, we do not consider them in our work.

Second restriction of the linear analysis concerns the period of the stable spatial structure formed. The system’s behavior depends strongly on the exact form of the nonlinear terms and on the shape and size of the reaction volume, so the period of the structure can not be determined percisely, but only as a range, depending on on the initial conditions. The system behavior strongly depends on the exact form of the nonlinear terms and on the shape and size of the reaction volume.

Finally, the sufficient conditions for the Turing-type instability are difficult to formulate accurately for the systems of more than two equations. Detailed description of the analysis in general form can be found in the recent study [22], however in this work there is no acurate consideration of the wave-type instability case.

### Computational approaches for integrating the reaction-diffusion equations

Simulation of the dynamic system based on the equations with partial derivatives implies simultaneous integration over space and time. Computational methods used for numerical solution of these equations are well known but are rarely mentioned in context of pattern’s formation simulation. Here, in order to fill this gap, we provide the complete description of the algorithm, as well as the source code based on it.

For a numerical solution the method of splitting with respect to physical processes is used. The kinetic equations are solved by the Runge-Kutta method, while the diffusion equations are solved using alternating directions implicit (ADI) method. In case when the kinetic rate constants are big (systems 3 and 4 in Fig 8b) the following Rosenbrock stiff scheme is used instead of Runge-Kutta method:

$$\begin{array}{c} \left(E-a\tau J-b\tau^{2}J^{2}\right)\frac{{\vec{x}}_{n+1}-{\vec{x}}_{n}}{\tau }=\vec{f}\left({\vec{x}}_{n}+c\tau \vec{f}\left({\vec{x}}_{n}\right)\right), \end{array}$$

where J is the Jackobian of the vector-function *f* and *a*, *b*, *c* are parameters set to 1.077, -0.372, -0.577 respectively [23, 24].

Two-dimensional diffusion problem was reduced to two one-dimensional problems by ADI and then was solved using the so-called “flux sweeping” method (see S2 Appendix) [25]. All algorithms are implemented as C++ functions and were run at high performance machines. All concentration distributions, as well as other plots are built with python using libraries *numpy*, *scipy* and *matplotlib*.

### Ethics statement and biological illustrative material

*Xenopus laevis* embryos were obtained by artificial fertilization and staged according to [26]. *Synodontis multipunctatus* fries were purchased in a pet shop. The whole-mount *in situ* hybridization was done as described in [27] with dig-labeled probe synthesized by T3 polymerase from pCS2-Sox2 (gift of T. Sasai) cut by BamHI.

Animal experiments performed in this study were specifically approved by Shemyakin-Ovchinnikov Institute of Bioorganic Chemistry (Moscow, Russia) Animal Committee in accordance with the guidelines and handled in accordance with the Animals (Scientific Procedures) Act 1986 and Helsinki Declaration.

## Results

### Turing-type instability conditions for the classical Gierer-Meinhardt model

The classical GM model in its simplest form is formulated as follows [10]:

$$\left\{\begin{array}{cc} \frac{\partial u}{\partial t} & =\rho \frac{u^{2}}{v}-\mu_{u}u+D_{u}\Delta u,\\ \frac{\partial v}{\partial t} & =\rho u^{2}-\mu_{v}v+D_{v}\Delta v. \end{array}\right.$$ (1)

Here *u* demonstrates autocatalytic behavior and is called “activator”, while *v* hinders the activator autocatalysis and is called “inhibitor”. *D*_*u*_, *D*_*v*_ are diffusion coefficients, *ρ* is production rate, *μ*_*u*_, *μ*_*v*_ are degradation rates.

**Fig 2:**
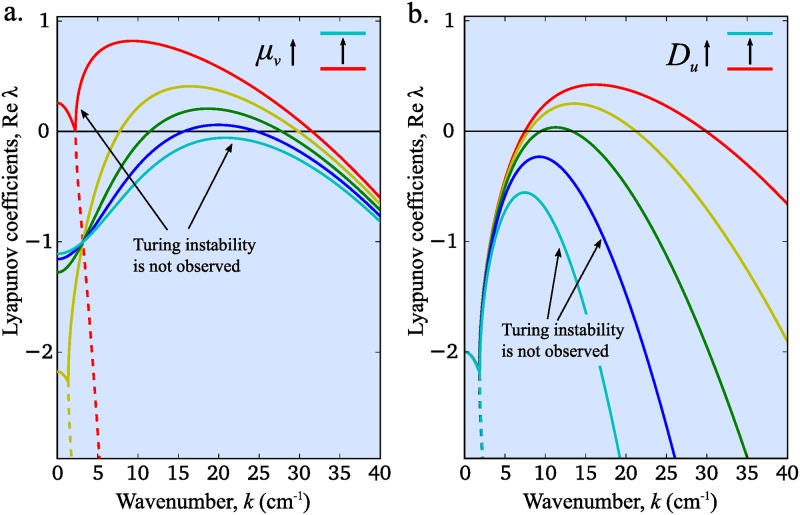
Dispersion curves for various inhibitor degradation rates (a) and for various activator diffusion rates (b) in classical GM model. Dispersion curves are plotted using formula obtained in Eq (S1.2.6). Following parameters are used in both panels: *μ*_*u*_ = 1, *D*_*v*_ = 0.1. In panel (a): *D*_*u*_ = 0.001 and *μ*_*v*_ = 0.5, 5.4, 10.3, 15.3, 20.0 (colored by rainbow from red to cyan). In panel (b): *μ*_*v*_ = 5 and *D*_*u*_ = 0.001, 0.0018, 0.0032, 0.0056, 0.01 (colored in the same way).

In Fig 2a we indicate the range of wave numbers that can provide formation of stable homogeneous structures for different values of the inhibitor degradation rate (*μ*_*v*_). Red curve corresponds to the minimal value of *μ*_*v*_ when the Turing instability does not occur. In the case of red curve reaction part is unstable (**Re** *λ*(0) > 0); this is another type of instablitiy which does not follow to pattern formation. Increasing *μ*_*v*_ we obtain a wide range of wave numbers with positive real parts corresponding to possibility of pattern formation (yellow curve). This range is narrowing with further increase of *μ*_*v*_ and finally damps (cyan curve). Thus, the regime of stable pattern formation in system (1) can be controlled by changing the value of the inhibitor degradation rate. If we then fix all the reaction constants and focus on the diffusion terms one can notice that variation of the activator diffusivity also affects the possibility of pattern formation. Indeed, while we observe a wide range of unstable waves for small values of *D*_*u*_ (Fig 2b, red curve), increase of its value leads to increase of the pattern periodicity until finally the Turing instability disappears (Fig 2, blue and cyan curves).

The analytical necessary and sufficient conditions of the Turing instability in system (1) are relatively simple and have already been formulated in several papers [28, 29]. They have a form of two inequalities, which link the diffusion and the degradation parameters (see S1.2 for derivation):

$$\{\begin{array}{l} \mu_{v}>\mu_{u},\\ \frac{D_{v}}{D_{u}}>\frac{\mu_{v}}{\mu_{u}}\frac{1}{3-2\sqrt{2}}. \end{array}$$ (2)

Thus, Turing instability in classical GM models arises only for particular range of both diffusion and reaction rates (Fig 2). Furthermore, as it follows from Eq (2) even small difference between *μ*_*v*_ and *μ*_*u*_ implies sixfold difference between the diffusion coefficients. The conventional condition of the Turing-type instability in system (1) is a sharp difference in the diffusion coefficient values: *D_u_* << *D_v_*. However, proteins involved in morphogenesis in embryo have approximately the same molecular size and thus very close coefficients of free diffusion. As the result, pattern formation for such system cannot be described with classical GM model. In the next section we propose an alternative mechanism of pattern formation for the case of close diffusion coefficients of reactants.

### Introduction of the morphogen adsorption into the two-component model

Let us consider a system of two morphogens *u* and *v* with same value of diffusion coefficients: *D*_*u*_ = *D*_*v*_ = *D*. In order to take into account adsorption of morphogens on ECM we add the following reaction between the activator and the virtual binding centers of HSPG, *W*:

$$\begin{array}{c} U+W⇆B \end{array}$$

Then, our extension of model (1) can be written as follows:

$$\begin{array}{c} \left\{\begin{array}{cc} \frac{\partial u}{\partial t} & =\rho \frac{{\left(b+u\right)}^{2}}{v}-\mu_{u}u-k_{1}wu+k_{-1}b+D\Delta u,\\ \frac{\partial v}{\partial t} & =\rho {\left(b+u\right)}^{2}-\mu_{v}v+D\Delta v,\\ \frac{\partial w}{\partial t} & =-k_{1}wu+\left(k_{-1}+\mu_{u}\right)b. \end{array}\right. \end{array}$$ (3)

Here *u* and *v* are concentrations of free morphogens playing roles of activator and inhibitor respectively, *w* is the concentration of the available binding sites on ECM. Parameters *ρ*, *D* and *μ*_*u*_, *μ*_*v*_ retain their meaning from Eq (1), *k*_1_ is the rate of adsorption of activator on ECM and *k*_−1_ is the rate of inverse reaction. *b* = *w*_0_−*w* is the concentration of morphogen-activator adsorbed to ECM, where *w*_0_ = *w*(*x*, 0).

**Fig 3:**
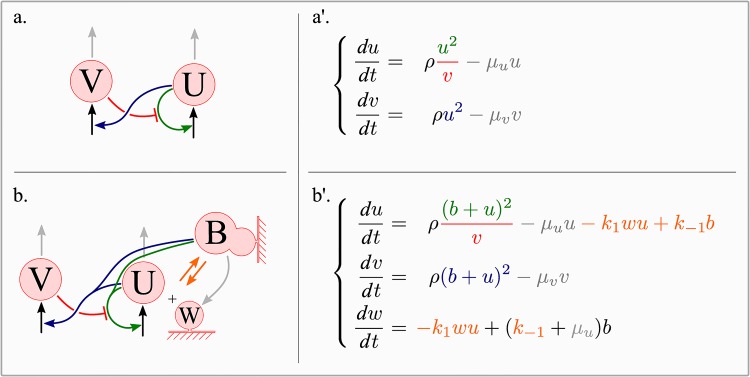
The derivation of the extended Gierer-Meinhardt model. Kinetic scheme for the classical (a) and the extended (b) GM models are presented on the left side; the corresponding equations are on the right. Arrows indicating interactions between reactants have the same color as the reaction terms in corresponding equations: green for autocatalytic terms, red for inhibition of activator by inhibitor, gray for degradation terms and orange for the adsorption and the desorption terms.

In our model morphogen-activator supports autocatalysis and activates morphogen-inhibitor (*v*) in both free (*u*) and adsorbed (*b*) states. We assume that the rate of degradation of morphogen-activator in adsorbed state as the same as for free activator and equals *μ*_*u*_. As a result, free binding sites appear due to both desorbtion of morphogen (*k*_−1_*b*) and its degradation in adsorbed state (*μ*_*u*_*b*). All the reactions considered in GM model (1) and in our extension of GM model (3) are summarized in (Fig 3) with arrows having the same color as corresponding reaction terms.

Let us then compare the conditions on stable pattern formation in system (1) and in system (3) in case of homogeneous distribution of binding centers.

### Turing-type instability conditions in the extended model

One can demonstrate that Turing-type instability arises in system (3) if and only if the following conditions are satisfied (see S1.3 for their derivation):

$$\begin{array}{c} \left\{\begin{array}{c} \mu_{v}>\mu_{u},\\ \lambda_{1}\in R,\;\lambda_{1}>0,\\ \text{Re}\;\lambda_{2,3}<0, \end{array}\right. \end{array}$$ (4)

where *λ*_1,2,3_ are eigenvalues of the matrix:

$$\begin{array}{c} A=\left(\begin{array}{ccc} \mu_{u}-k^{2}D & -\mu_{u}^{2}/\rho & -k^{2}D\\ 2\rho \mu_{v}/\mu_{u} & -\mu_{v}-k^{2}D & 0\\ -k_{1}\bar{w} & 0 & -k_{-1}-k_{1}\left(\bar{u}+\bar{w}\right)-\mu_{u} \end{array}\right), \end{array}$$

where $\bar{u},\bar{w}$ are stationary solutions of the kinetic system and *k* is wave number. Note, that condition on the degradation rates in Eq (4) is the same as the condition derived for the classical GM model (2). Below we demonstrate that in general extended GM model (3) retains many features of the classical GM model (1).

**Fig 4:**
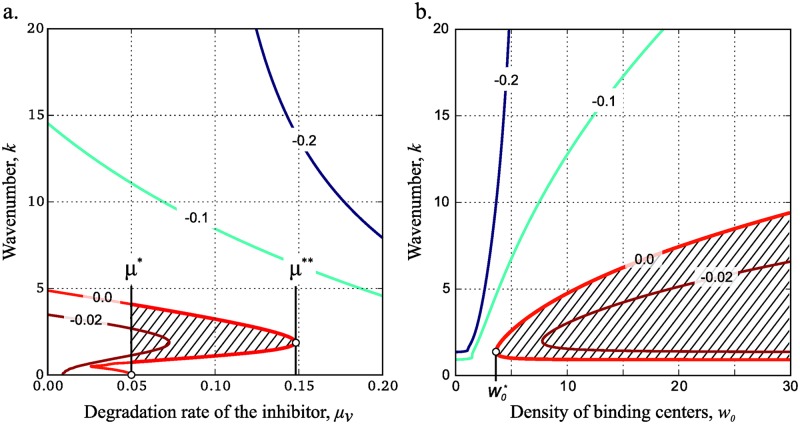
Turing instability for different values of *w*_0_ and *μ*_*v*_. Level lines of {max **Re** *λ*(*k*)} for different values of parameters and wave number (see supplementary equation (S1.3.9) for details). By definition, any vertical cross-section of the map gives a dispersion curve (similar to Fig 2). Dashed area indicates parameter values for which real parts of Lyapunov coefficients are positive and thus Turing instability can occur. The following model parameters were used for the development of the maps: *k*_1_ = 1, *k*_−1_ = 0.1, *D* = 0.1, *ρ* = 0.6, *μ*_*u*_ = 0.05 and *w*_0_ = 7 (a) or *μ*_*v*_ = 0.08 (b).

In order to compare behavior of system (3) with that of system (1) let us divide model parameters into two groups: the “reaction” parameters that have the same sense in both systems (*ρ*, *D*, *μ*_*u*_ and *μ*_*v*_), and the “adsorption” parameters used only in the extended model (*w*_0_, *k*_1_ and *k*_−1_). Influence of the inhibitor degradation rate (*μ*_*v*_) on the dispersion curve behavior is very similar for both classical GM (Fig 2a) and extended (Fig 4a) models. In system (3) Turing instability occurs for the values of *μ*_*v*_ in the interval (μ*, μ**) (Fig 4a).

Let us then consider the role of adsorption parameters in model (3). Affinity of activator to the surface is stronger with increase of *w*_0_ or *k*_1_ and decrease in *k*_−1_. Kinetic adsorption parameters (*k*_1_, *k*_−1_) are determined for particular molecules, whereas *w*_0_ can vary depending on the expression level of the corresponding molecules in embryo. Consequently, *w*_0_ must crucially affect the process of pattern formation in system (3). The effect is observed in Fig 4b and is in accordance with the behavior of classical GM model. Indeed, in system (1) the necessary condition on Turing-type instability implies significant difference in the values of diffusion coefficients. Parameter *w*_0_ in system (3) regulates “adsorption strength” and thus provides the necessary difference for the rates of effective diffusion. As the result, in the extended GM model Turing-type instability appears for densities of binding sites above the critical value $w_{0}^{*}$ that corresponds to the abscissa of the vertex of the level line max Re *λ* = 0 (Fig 4b, red line).

We show below that the Turing instability appears when the measure $\frac{k_{1}w_{0}}{\mu_{u}+k_{-1}}$ (which stands for an effective diffusion coefficient of the activator) reaches the threshold value.

### Application of the extended GM model to natural systems

Validation of patterning schemes obtained in dynamic systems with Turing-type instability is restricted by the number of the model parameters available from the experiment. Since reaction rates are determined for the fixed reagents, formation of stable patterns is controlled by the coefficients of effective diffusion. As it was mentioned above, in the particular case of pattern formation in morphogenesis, coefficients of free diffusion of activator and inhibitor are estimated to be very close and thus the difference in effective diffusion rates must result from other system properties. While such behavior cannot be reproduced using classical GM model, our extended GM model operates biologically relevant parameters that can affect effective diffusion rate: adsorption/desorption rates and binding sites’ density. Each of these parameters can be determined and regulated separately in experiments. Below, we present three examples of different patterning events and demonstrate how our extended model can be used to analyze these processes.

#### WNT/DKK hair follicle patterning

**Fig 5:**
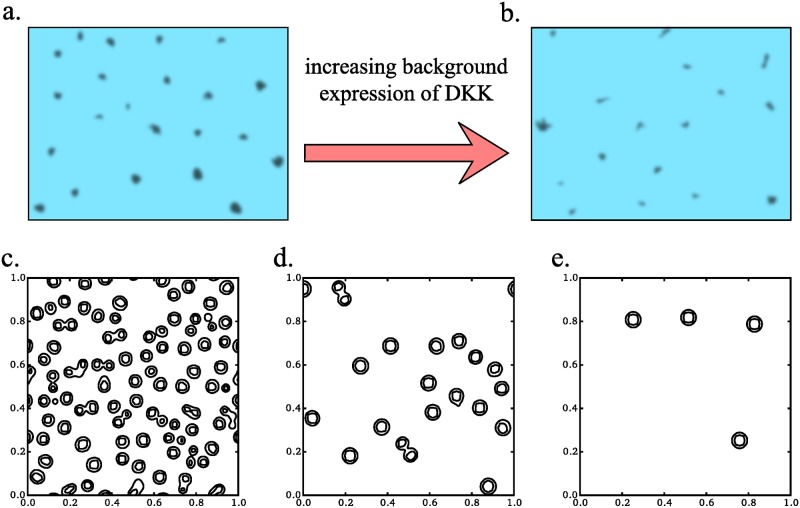
Simulation of follicle formation by using the extended GM model. WNT activation pattern in wild type (a) and DKK+ transgenic mice (b) redrawn from [30]. Turing structures formed from homogenious noise in 5 minutes (internal time) with different levels of base DKK expression: 0.1, 0.15 and 0.2 *μ*M/s (c-d). Other parameters used are shown in Table 1.

According to the recent study [30], during the follicle pattering, WNT and DKK act as activator-inhibitor system (WNT acts as activator, and DKK acts as inhibitor). The role of the base level of DKK expression in this process was estimated by comparison of follicle patterns in wild-type mice and in transgenic mice with the additional *Dkk* gene under skin-specific promotor. The similar effect of expression levels of DKK on pattern formation was reproduced in the mathematical model. However, in the original paper model parameters were chosen arbitrary and thus do not correspond to the experimentally measured constants. In particular, 40-fold difference in the values of diffusion coefficients for WNT and DKK introduced by the authors is hardly relevant. According to approximate equations based on WNT and DKK protein sizes [31], the value of diffusion coefficient of WNT is only about 20% lower than that of DKK. This discrepancy can be avoided is one takes into account adsorption of Wnt protein on ECM. Below we use our model (3) to reproduce WNT patterning and fit the parameters with physically consistent constants. Additional base production of the inhibitor was added to the second equation as *ρ*_*v*_. Increasing this parameter we imitates increase of constant expression of DKK in transgenic mice. Structures with high level of WNT after simulation along 5 minutes are shown at Fig 5c–5e. As we can see the density of structures is decreased with increase of base DKK expression as it was shown in the experiment (Fig 5a and 5b). Thus, our model can reproduce results of author’s experiments and modeling. At the same time using our model we are able to address almost every parameter value.

We set free diffusion coefficient *D* to 100 *μm*^2^/*s* that is close to hydrodynamic estimates for both WNT and DKK proteins [31]. Values of adsorption and desorption rate constants of WNT were never measured. At the same time, it is known that WNT adsorbtion on cell surface is increased due to the presence of palmitoyl group [32] and due to the ability of WNT to bind HSPG [33]. Thus, we choose *K*_*d*_ = 0.025 *μ*M that is less than *K*_*d*_ constants for other heparin-binding proteins (0.1–1 *μ*M [17, 34]). Direct and reverse rate constants were selected to fit an equation *k*_1_/*k*_−1_ = *K*_*d*_.

Clearance rate constants *μ*_*u*_, *μ*_*v*_ were measured in embryos only once for Lefty/Nodal proteins [28]. Authors obtained constants of order 10^−4^ *s*^−1^ however they did not take into account possible protein inactivation. Here we used constants of order 10^−3^ *s*^−1^ that is also consistent value. It means that average protein lifetime in the intercellular space is about several minutes.

The density of binding sites was also never measured in experiment. However, for the particular system concentration of binding sites can be converted to surface density if one knows the thickness of intercellular space. Indeed, *in vivo* reaction takes place in thin layers of intercellular spaces of about 0.2 *μ*m. Hence, we can assume that *w*_0_ = 10 *μ*M corresponds the density of 6 sites per 100×100 nm square. The estimate seems to be consistent and does not contradict current data on ECM.

Finally the working range of concentrations along the patterning reaction are in micromolar range. Recently we have demonstrated that proteins may be concentrated at micromolar level [16] in the intercellular spaces of *Xenopus* ectoderm, thus our estimate is also consistent.

The simulation parameters as well as the resulting concentration ranges are summarized in Table 1.

**Table 1:** Parameters of the extended GM model and their values fitted for reproducing Wnt/DKK hair follicle patterning.

Notation	Value	Explanation [*measure units*]
*ρ*	0.05	Empirical parameter characterizing strength of Wnt-induced Wnt and DKK production [*a.u.*].
*μ*_*u*_	0.003	Wnt degradation rate [*s*^−1^]
*μ*_*v*_	0.008	DKK degradation rate [*s*^−1^]
*D*	100	Free diffusion coefficient [*μm*^2^ ⋅ *s*^−1^]
*k*_1_	7.8	Adsorption rate constant [*μM*^−1^ ⋅ *s*^−1^]
*k*_−1_	0.2	Desorption rate constant [*s*^−1^]
*w*_0_	10	Volume binding site density [*μM*]
*u*(*t*)	0–0.1	Non-bonded WNT fraction [*μM*]
*u*(*t*) + *w*_0_ − *w*(*t*)	0–50	Total WNT fraction [*μM*]
*v*(*t*)	0–50	DKK concentration [*μM*]
*ρ*_*v*_	0.02–0.03	Permanent independent production of DKK [*μM* ⋅ *s*^−1^]

#### Patterns with spatially-graded spot size

**Fig 6:**
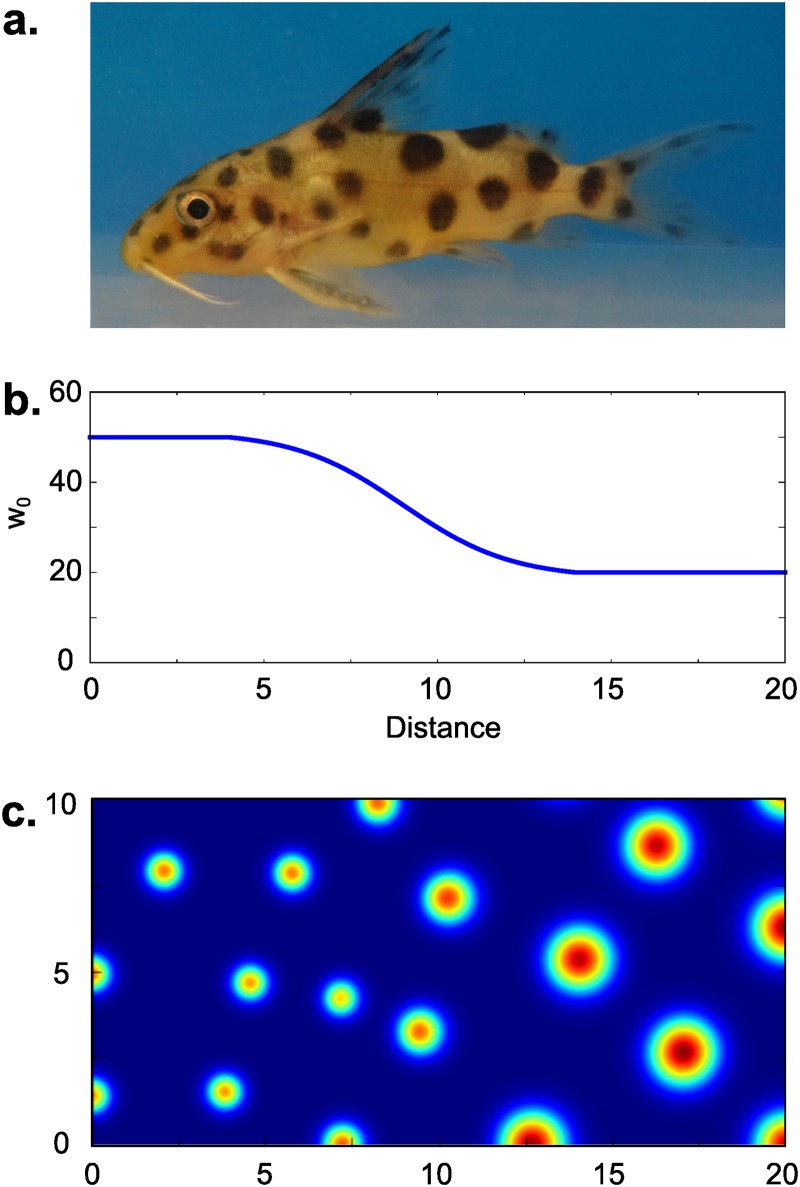
Spot patterning with gradient of spot sizes. Spots of various size are common for fish patterning, e.g. juvenile of *Synodontis multipunctatus*, that has spots of increasing size from tail to head (a). We have reproduced this pattern using model (3) with corresponding function *w*_0_(*x*) (b). The resulting pattern formed by *u* is given in (c) for *t* = 8000. Model parameters are: *k*_1_ = 0.97, *k*_−1_ = 0.1, *ρ* = 0.6, *μ*_*u*_ = 0.03, *μ*_*v*_ = 0.08, *D* = 0.1.

The choice of non-homogeneous distribution of binding site density (*w*_0_) in the extended model (3) allows us to reproduce patterning types that occur in nature. Here we take as an example establishment of the cuckoo catfish *Synodontis multipunctatus* coloring pattern. Cuckoo catfish juveniles are colored with spots of the increasing size from head to tail (Fig 6a). Let us consider this type of pattern in terms of model (3). For each *w*_0_ greater than $w_{0}^{*}$ Turing instability occurs for the range of wave numbers (*k*_*L*_, *k*_*R*_), where *k*_*L*_ and *k*_*R*_ are left and right critical wave numbers respectively (Fig 4b). Left critical wave number regulates the average period of the spotty pattern, whereas right critical wave number determines the size of a single spot. In our model increase of *w*_0_ leads to increase of *k*_*R*_, while almost does not affect *k*_*L*_ (Fig 4b). Thus, considering *w*_0_(*x*) to have smooth gradient form (Fig 6b) in model (3) we can reproduce pattering scheme with increasing spot size close to the skin pattern of the catfish juvenile (Fig 6c).

In contrast to zebrafish, catfish’s spot-patterning is formed due to difference in size of melanocytes [35], which in turn can be regulated by secreted substances: melanin-concentrating and melanocyte-stimulating hormones. It is shown that melanin-concentrating and alpha-melanocyte stimulating hormones have an antagonistic behavior on skin patterning in teleost [36]. Unfortunately, the mobility of these factors and their interactions with matrix are studied insufficiently, which makes our hypothesis impossible to be proved with any quantitative estimations at present time.

#### Pear shape of the neural anlage

**Fig 7:**
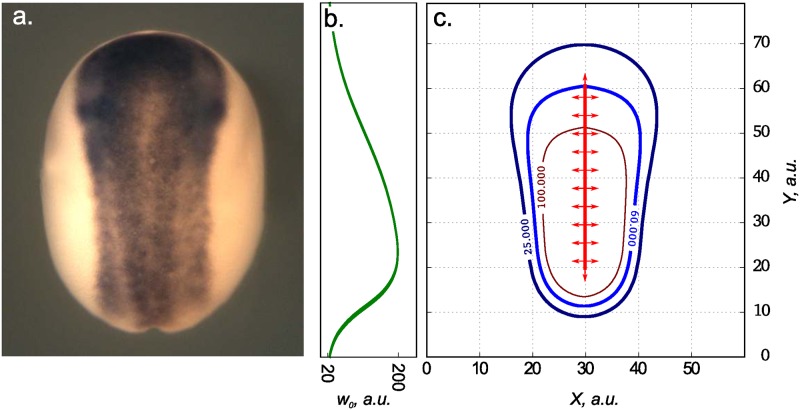
Modeling of the neural plate’s shape in the early *Xenopus laevis* gastrula. The neural plate at the *X. laevis* midneurula visualized by the whole-mount *in situ* hybrydization with dig-labeled probe to the pan-neural marker, *Sox2* (a). Distribution of HSPG sites in ECM used as *w*_0_(*x*) for simulation of model (3) (b). Level curves for stable structures formed by activator (Chordin) in the simulation of model (3) for *t* = 1000 (c). Model parameters are: *k*_1_ = 1.0, *k*_−1_ = 0.01, *ρ* = 0.6, *μ*_*u*_ = 0.01, *μ*_*v*_ = 0.05, *D* = 0.1.

Let us consider another pattern that can be reproduced using model (3). In *X. laevis*, neural plate has a shape of a pear with wide and narrow parts in the anterior and posterior regions respectively (Fig 7a). The size of the neural plate is sensitive to changes in the overall ectoderm size (till the late neurula) [37], hence the system is robust. It is known that the robustness to parameters, concentrations and volume fluctuations is very common feature for Turing structures [38]. Thus, formation of pear-shaped neural anlage may be considered as the result of Turing structure formation.

The process of tissue self-organization implies complex interplay between many secreted proteins. In case of *X. laevis* neural plate, key role is played by Bone Morphogenetic Protein (BMP) that forms a ventral to dorsal gradient. Formation of neural tissue appears as the result of BMP inhibtion by antagonists such as Chordin and Noggin [39–41], that propagate from the dorsal midline through IS and via the Brachet’s cleft [42]. In the absence of Chordin and Noggin all the embryonic ectoderm differentiates to epidermis under action of BMP. Thus, spatial distribution of concentration of Chordin and Noggin determines the shape of the neural plate.

Chordin is known to increase its own secretion through the inhibition of BMP cascade [39], which also leads to the increased expression of anti-dorsalizing morphogenic protein (ADMP). ADMP in turn inhibits Chordin expression through the activation of BMP cascade [43]. Thus, one can consider interaction of Chordin and ADMP as activator-inhibitor system as it was done in models of *X. laevis* dorso-ventral patterning [2, 19].

Let us consider model (3) with *u* being concentration of Chordin constantly produced from the dorsal midline and *v* being concentration of ADMP. Chordin has heparin binding motifs and thus its diffusion is retarded by its intercation with HSPG of ECM [16]. According to our recent study, concentration of HSPG within the ectoderm of the *X. laevis* embryo during gastrulation and neurulation forms a gradient with maximum at the dorsal posterior (presumptive tail) and minimum at the anterior (presumptive head) region [17]. Using corresponding distribution of binding sites *w*_0_(*x*) (Fig 7b) in our model (3), we obtain stable non-homogeneous distribution of activator that resembles a real pear-like shape of the neural plate (Fig 7c).

### Transformation of the extended model to the classical one: Effective diffusion concept

Both classical and extended GM models can be applied for the description of patter formation in activator-inhibitor system. Extended model (3) is appropriate for the description of pattern formation in case of similar size of reactants and significant role of adsorbtion of activator on some immobile substance. As we have demonstrated above, these features are common for different systems regulating the process of morphogenesis. However, for some biological systems of that type the use of extended model is not reasonable and it can be reduced to the classical GM model. Here we describe reduction of system (3) to system (1) using quasi-steady state approximation and the formalism of effective diffusion.

#### Necessary conditions for model reduction

First, adsorbtion of activator on ECM can be neglected only if the diffusion process is slower than the binding process. In term of our models, this condition can be formulated as follows:

$$\begin{array}{c} \frac{1}{w_{0}k_{1}}<<\frac{1}{k^{2}D},\;\;\frac{1}{k_{-1}}<<\frac{1}{k^{2}D}. \end{array}$$

The second condition concerns adsorption mechanism. Binary adsorption is commonly described by Langmuir isotherm: *θ* = *KC*/(*KC*+1), where *θ* is the ratio of bound and unbound centers, *K* is association constant and *C* is concentration of the adsorbate. When concentration of adsorbate is small with respect to the concentration of adsorbent, we can use the linear isotherm: *θ* = *KC*. Thus, the second condition requires isotherm to be linear and, consequently, the density of binding sites to be large:

$$\begin{array}{c} w_{0}>>0\;. \end{array}$$

These conditions describe so called “effective diffusion behavior” (the terminology suggested by S. McNally [44]). This behavior was introduced by J. Crank in chapter “Instantaneous reaction” of his well-known book [45]. If the binding reaction affects the overall process in the same way as a decrease in the diffusion coefficient does, then “the effective diffusion coefficient” could be used and calculated as following:

$$\begin{array}{c} D_{eff}=\frac{D}{1+\frac{k_{1}w_{0}}{k_{-1}}}\;. \end{array}$$ (5)

Accordingly, in our case very close estimation of the effective diffusion coefficient can be formulated.

#### Reduction of the equations to none-adsorption model

Suggest adsorption of the activator tends to infinity (*w*_0_, *k*_1_, *k*_−1_ → ∞) and simultaneously $\frac{w_{0}k_{1}}{k_{-1}+\mu_{u}}=const$; then the system (3) can be reduced to Eq (1) with

$$\begin{array}{c} D_{v}=D,\;D_{u}=D\cdot {\left(1+\frac{w_{0}k_{1}}{k_{-1}+\mu_{u}}\right)}^{-1}, \end{array}$$

and

$$\begin{array}{c} \rho_{red}=\rho {\left(1+\frac{w_{0}k_{1}}{k_{-1}+\mu_{u}}\right)}^{3/2}. \end{array}$$

*D*_*u*_ found by this equation is called “effective diffusion coefficient”. As we follow J. Crank’s line of evidence [45]. We first should rewrite Eq (3) assuming that the adsorption reaction is extremely fast and *w*, *u* and *w*_0_ are in local equilibrium at each point:

$$\begin{array}{c} \left\{\begin{array}{cc} \frac{\partial u}{\partial t} & =\rho \frac{{\left(u+w_{0}-w\right)}^{2}}{v}-\mu_{u}\left(u+w_{0}-w\right)+\frac{\partial w}{\partial t}+D\Delta u,\\ \frac{\partial v}{\partial t} & =\rho {\left(u+w_{0}-w\right)}^{2}-\mu_{v}v+D\Delta v,\\ 0 & =-k_{1}wu+\left(k_{-1}+\mu_{u}\right)\left(w_{0}-w\right). \end{array}\right. \end{array}$$ (6)

In our initial approximation *w* > >*u* and, thus, the term *k*_1_*w* could be replaced with *k*_1_*w*_0_. Consequently:

$$\begin{array}{c} w_{0}-w\approx \frac{k_{1}w_{0}}{k_{-1}+\mu_{u}}u \end{array}$$

We denote adsorption coefficient $\frac{k_{1}w_{0}}{k_{-1}+\mu_{u}}$ as *α*. Using this equation let us remove *w* from the two remaining equations of system (6):

$$\begin{array}{c} \left\{\begin{array}{cc} \frac{\partial u}{\partial t} & =\rho {\left(1+\alpha \right)}^{2}\frac{u^{2}}{v}-\mu_{u}\left(1+\alpha \right)u-\alpha \frac{\partial u}{\partial t}+D\Delta u,\\ \frac{\partial v}{\partial t} & =\rho {\left(1+\alpha \right)}^{2}u^{2}-\mu_{v}v+D\Delta v. \end{array}\right. \end{array}$$

Finally we replace *u* = u*/(1+*α*) and get:

$$\begin{array}{c} \left\{\begin{array}{cc} \frac{\partial u^{*}}{\partial t} & =\rho \frac{u^{*2}}{v}-\mu_{u}u^{*}+\frac{D}{1+\alpha }\Delta u,\\ \frac{\partial v}{\partial t} & =\rho u^{*2}-\mu_{v}v+D\Delta v. \end{array}\right. \end{array}$$ (7)

Eq (7) has the same form as Eq (1).

**Fig 8:**
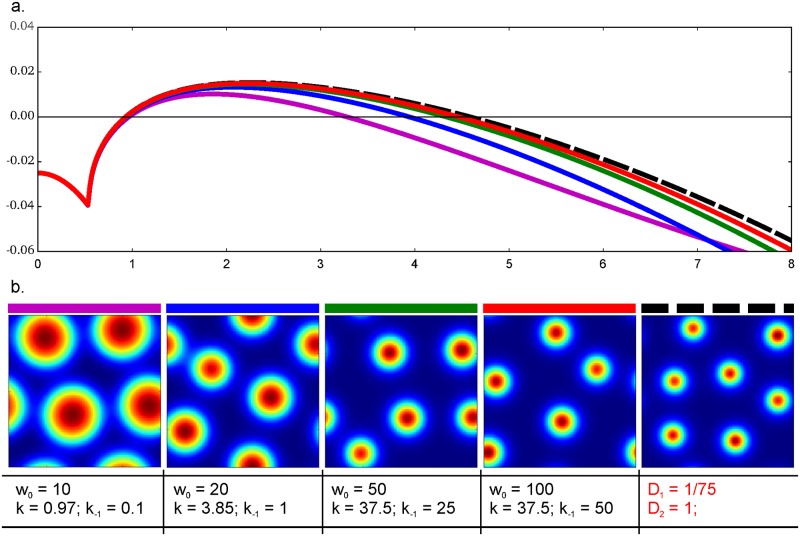
Reduction of the three-component model to the two-component one. A: Dispersion curves of the extended model (solid lines) and the conformable classical model (dashed line). Eq (7) were used for the classical model. For each curve parameters of adsorption are presented under corresponding images **B**. Other parameters are fixed for both extended and classical models: *ρ* = 0.6, *μ*_*v*_ = 0.08, *μ*_*u*_ = 0.03. Reaction space of 20×20 space units and Neumann boundary conditions were used. Size of the reactor was set as 20 units. B: Concentration of activator as visualized after 4000 time units of the simulation with the above mentioned parameters.

As the assertion predicts, dispersion curves of the extended system tend to dispersion curves of the reduced system (7). Varying *α* we affect Turing structures of the extended model in the same way as changing *D*_*u*_ affects structures in the classical model (Fig 2b). If we fix the ratio $\frac{k_{1}w_{0}}{k_{-1}+\mu_{u}}$ and draw the family of dispersion curves (Fig 8a), then the better similarity between dispersion curves of the reduced and the extended models is observed under high values of *w*_0_ and rate constants *k*_1_, *k*_−1_ (green and red curves). Although the proximity of dispersion curves points at the similarity in linear regimes of these two models, the shape and size of the final Turing structures can be different for the extended and reduced systems. Under unfavourable conditions (in magenta) Turing structures of the extended system are larger then those in the reduced one (Fig 8b). In our case Turing structures demonstrate the behavior that corresponds to the behavior of dispersion curves: under conditions favourable for the reduction (in red) Turing structures have the same structure in both augmented and reduced systems.

## Discussion

Modelling of pattern formation with physically relevant parameters is the subject of many modern studies on embryogenesis. There are three major studies on early patterning, that consider *Xenopus laevis* embryo as an example. In the most thorough of them, that describes by Turing model the left-right patterning [28], the authors measured independently almost each parameter. Another article with a quite demonstrative base describes dorso-ventral gradient [18], however the authors were far from measuring every parameter independently. One more model without substantial proof was suggested earlier by Meinhardt himself [46]. The diffusion coefficients used in all of these works differ from each other not less than an order of magnitude. While the authors of the cited works did not specially explain the latter fact, one may suppose that such a big difference in diffusivity between morphogens having the comparable sizes could be explained only by a difference in their adsorption on the cell surface. Thus, inclusion of the conditiion of the morphogens adsorption on ECM into the equations during the modeling of embryonic patterning is an important task.

In the present work, we have developed a model, which describes self-organization of the long-range spatial structures in conditions of adsorption of one of the reacting components on ECM. Many secreted proteins can adsorb on ECM due to electrostatic interactions of specialized motifs in their content with heparin moieties in HSPG. For example, heparin-binding motifs were found in such morphogens as Activin, BMP, Cerberus, Chordin, the member of EGF and Fgf families, Nodal, Noggin1 and Noggin2. Because of these interactions, the diffusivity of morphogens in embryonic tissues is usually estimated by values 10–100 less than one may expect for freely diffusing proteins. Therefore, a consideration of the morphogens adsorption on ECM as an important factor influencing diffusion seems to be wholly justified when modeling embryonic patterning.

The extended Gierer-Meinhardt model (as well as any adsorption modified patterning model) can be applied in biological systems. We probe it on the well known WNT-DKK patterning system describing hair follicle formation. This model fits to the experimental data using physically considerable parameter values and, thus, describes embryonic patterning at quantitative level. Moreover, our theory allows us to make preliminary estimations even now on the base of indirect data. Indeed, according to the literature, the affinity of various organic polymers, including some protein morphogens, to HSPG varies in a range from 0.1 to 1 *μ*M [47]. Suggest the effective diffusion coefficients of many morphogens measured in live embryonic tissues usually appear in 10–100 times lower than those which could be predicted for the same morphogens on basis of hydrodynamic laws; then according to the Eq (5), the density of binding sites may be estimated as 10–100 *μ*M. This indicates that the density of binding sites in intracellular space is several orders of magnitude larger than maximal concentration of morphogens, that usually doesn’t exceed several nanomoles. Obviously, special experiments should be arranged to determine values of all these parameters precisely in each real embryonic tissue.

As we shown, the addition of the adsorption term to the Gierer-Meinhardt model, significantly extends the spectrum of spatial structures, which potentially can be obtained on the base of the original model. We can simply form the most interesting extension of the model keeping values of all other parameters and forms of equations together with varying the concentration of the adsorption sites, i.e. by changing the concentration of HSPG in the IS. It seems logical to suppose that such mechanism of the patterning regulation could be widely utilized during the embryonic development. Indeed, HSPG in IS of the embryonic tissues are frequently distributed unevenly, forming gradients in selected directions. In particular, HSPG forms the dorsal to ventral gradient with the maximum on the dorsal marginal zone in the ectoderm and the mesoderm of the *Xenopus laevis* embryo during gastrulation and neurulation [17]. As a result, a decreased concentration of potential adsorption sites for morphogens is revealed within the presumptive anterior neural plate comparing to the more posterior presumptive trunk region. Accordingly, as we have showed, this may result in spreading of the neural inductors over more broad territory in the anterior region and lead to formation of the pear-shaped form of the neural plate. Certainly, the patterning of neural anlage involves greater number of agents than we consider in the model. Nevertheless, we believe that our approach could be considered as a simple proof of principle of much more complex processes that underlay the neural plate patterning.

Another example of the transformations of the spatial pattern, which may be explained by the gradual changes in the concentration of the adsorption sites, can be the spatial changes frequently observed in coloring of fishes. Here, we consider only one example of such coloring that is implemented in fishes Fig 6a. In different species the size of colored spots and the distance between adjacent spots are progressively changing either from the dorsal to ventral side, or from the tail to the head. These patters resemble a lot those shown on Fig 6c, which was generated *in silico* by a virtual gradual decrease of the concentration of adsorption sites along the corresponding direction.

Earlier, the ventral side spot pattern of catfish *Plecostomus* have been simulated with simple two-component Turing-type model [35]. However, while the authors have demonstrated that Turing-type model well describes spotted pattern, they did not explain the fact of the gradual increase of the spot size. Our model expands this approach and suggests the mechanism of patterning with spots with gradually increasing size. The detailed study of fish pigmentation with identification of biological mechanism was performed hitherto only for *Danio rario*. It was shown that *Danio* pigmentation is robust [48] and can be described by Turing-type model of spatial activation of Delta-Notch pathway [49]. In contrast, catfishes are not being studied thoroughly and yet they have completely different pigmentation system. We believe that our work could stimulate further study that will experimentally demonstrate the role of the gradual changes of the adsorption in the fish coloring.

As we have recently shown, diffusion of a protein in conditions of its adsorption makes it impossible to use the diffusion coefficient as a single mobility parameter, because in this case the diffusivity of the protein can vary with protein concentration [16, 17]. That is why sometimes the diffusion coefficient measured by different authors with different techniques appears to be different. Moreover, researchers can determine different mobility constants for the single substance depending on the measurement conditions. This happens because the effective diffusion conditions could be satisfied under certain experimental conditions but could not under other ones. The present research allows us to understand whether the ‘diffusion coefficient’ makes sense or not. If we operate the effective diffusion constant, then we should be sure that the corresponding reaction-diffusion-with-adsorption system can be reduced to the reaction-diffusion system as we have shown in Eqs (6) and (7). Importantly, we have demonstrated that if the adsorption rate is higher than the rate of free diffusion and the number of binding centers for the protein adsorption is large, then a single constant parameter, the effective diffusion coefficient, can be used again. Thus, in such cases the extended model can be reduced to the classical form proposed by Gierer and Meinhardt.

For instance WNT-DKK patterning system can be reduced to the two-component model and single value of diffusion coefficient can be used. For this case the effective diffusion coefficient calculated by Eq (5) for the presented model is 0.25 *μ*m^2^/s whereas the same value measured with FRAP technique in *Xenopus* ectoderm is 0.08 *μ*m^2^/s [16] that is very close. In other cases one have to use three additional parameters in our extended version of Gierer-Meinhardt model instead of single diffusion coefficient to describe propagation of the activator: direct and reverse adsorption rate constants and binding site density. Obviously, to understand which variant of the model, extended or reduced, should be used in the selected case, one has to know the values of the aforementioned three parameters, namely direct and reverse adsorption rate constants and binding site density, in real living system. Measurement of these parameters experimentally become very perspective direction following from our studies.

The inclusion of adsorption as one of initial conditions in the reaction-diffusion system provides an exciting way for the Turing instability modulation, thereby expanding the possible specter of self-organizing structures. In our model, we introduced such modulation by varying binding site density which can vary in the real embryo along the selected direction. In a number of known models modulation of Turing instability is achieved by different mechanisms. For example, reaction-diffusion model with chemotaxis was used to simulate snake patterning and the chemotaxis strength changing along the snake body was used as Turing modulation parameter [50]. Note, that their model also described patterns with gradual changes in structure period. One more mechanism of Turing instability modulation was suggested by Sen [51]. This mechanism is based on varying the delay in delayed PDE. Biological evidences of modulating patterning also exist in the literature: for instance, in quail-duck chimera feather follicle patterning is modulated by concentration of BMP protein [52]. Apparently all these mechanisms occur in nature and produce all the observable diversity of embryonic patterns. We believe that further experimental investigations will shed light on biological mechanisms that underlay proposed models.

## Supporting information

S1 Appendix Linear analysis of Turing instability. Rigorous derivations of Eqs (2) and (4) are supplemented.
(PDF)

S2 Appendix Flux sweeping algorithm. In the appendix we perform description of flux sweeping computational scheme as reproduced from ref. [25] with adaptation to our case.
(PDF)
