# Supplementary material for: Morphogene adsorption as a Turing instability regulator: Theoretical analysis and possible applications in multicellular embryonic systems
Source: PLoS One. 2017 Feb 7;12(2):e0171212. doi: 10.1371/journal.pone.0171212 (PMC5295678; doi:10.1371/journal.pone.0171212)
Supplement: S1 Appendix — Rigorous derivations of Eqs (2) and (4) are supplemented. (PDF) [file pone.0171212.s001.pdf]

Full paper: Nesterenko A.M., Kuznetsov M.B., Korotkova D.D., Zaraisky A.G. Morphogene Adsorption on the Extacellular Matrix as a Possible Turing Instability Regulator in Multicellular Embryonic Systems. *PLoS ONE* (2017): 10.1371/journal.pone.0171212

Appendix S1. Linear analysis of Turing instability.

Appendix S1.1. General Turing conditions.

The necessary and sufficient conditions of the Turing instability arising in the systems of the reaction-diffusion equations are already known [1]. Let us consider the equation system has the following general form:

$$\partial_t \vec{c} = \mathbb{P}(\vec{c}) + \partial_{xx} \mathbb{D} \vec{c} \tag{S1.1}$$

Denote the stationary point by c_0:

$$\mathbb{P}(\vec{c_0}) = \vec{0}$$

Denote by $\mathbb{M}$ the Jacobian of the vector-valued function $\mathbb{P}$ at the point c_0:

$$\mathbb{M} = \left. \frac{d\mathbb{P}(\vec{c})}{d\vec{c}} \right|_{\vec{c}=\vec{c_0}}$$

For both two and three equations in the equation system (having a unique stationary point) the sufficient Turing conditions are the following:

1. **Reaction stability of the stationary point.** Matrix $\mathbb{M}$ should not have eigenvalues with a positive real part.
2. **Reaction-diffusion saddle-type instability of the stationary point.** Matrix $(\mathbb{M} - k^2\mathbb{D})$ should have only one eigenvalue with a positive real part at limited range of the wavenumber k.

Appendix S1.2. Turing instability analysis of the classical GM model

Here we provide the analysis of Classical Gierer-Meinhardt model defining by following equation:

$$\begin{cases} \dfrac{\partial u}{\partial t} = & \rho \dfrac{u^2}{v} - \mu_u u + D_u \Delta u \\ \dfrac{\partial v}{\partial t} = & \rho u^2 - \mu_v v + D_v \Delta v \end{cases} \tag{S1.2.1}$$

Firstly, the stationary point of the pure-reaction system $(\overline{u}, \overline{v})$ is solitary. We have

$$\begin{cases} \overline{u} = \mu_v / \mu_u \\ \overline{v} = \rho \mu_v / \mu_u^2. \end{cases} \tag{S1.2.2}$$

Reaction stability of the stationary point. The Jacobian matrix found at the stationary point, $\mathbb{M}$, is:

$$\mathbb{M} = \begin{pmatrix} \mu_u & -\mu_u^2/\rho \\ 2\rho\mu_v/\mu_u & -\mu_v \end{pmatrix} \tag{S1.2.3}$$

Eigenvalues of $\mathbb{M}$ are:

$$\lambda = 1/2\left(\mu_u - \mu_v \pm \sqrt{\mu_u^2 + \mu_v^2 - 6\mu_u\mu_v}\right)$$

This proves that *the reaction system* (S1.2.1) *is stable* ***iff*** μ_v *is bigger than* μ_u:

$$\mu_v > \mu_u \tag{S1.2.4}$$

It can be shown in the usual way, that the system will demonstrate oscillatory behavior when

$$(\mu_u - (3 - 2\sqrt{2})\mu_v)(\mu_u - (3 + 2\sqrt{2})\mu_v) < 0.$$

Finally, the first quarter of the parametric plane μ_u, μ_v is divided by three lines into the areas where different phase portraits are formed (Fig S1).

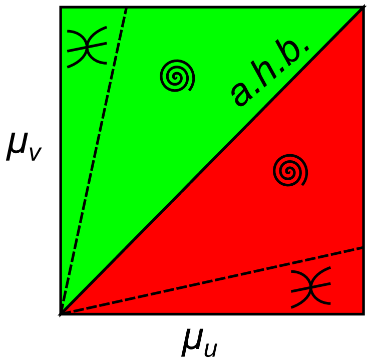

Fig S1. Bifurcation diagram of the system (S1.2.1) **in (μ_u, μ_v) parametric space.** The zone of stability of the stationary point is marked green, the opposite zone is marked red. Focus lost its stability when intersecting the diagonal line.

Reaction-diffusion saddle-type instability of the stationary point. The diffusion-augmented Jacobian matrix has the form:

$$\mathbb{M} - k^2\mathbb{D} = \begin{pmatrix} \mu_u - k^2 D_u & -\mu_u^2/\rho \\ 2\rho\mu_v/\mu_u & -\mu_v - k^2 D_v \end{pmatrix} \tag{S1.2.5}$$

Clearly, its eigenvalues:

$$\begin{aligned} \lambda_{1,2} = -1/2 \Big[& (k^2(D_u + D_v) + (\mu_v - \mu_u)) \pm \\ & \pm \sqrt{(k^2(D_u + D_v) + (\mu_v - \mu_u))^2 - 4(k^4 D_u D_v + k^2(D_u\mu_v - D_v\mu_u) + \mu_u\mu_v)} \Big] \end{aligned} \tag{S1.2.6}$$

Only one λ value have a positive real part **iff**

$$k^4 D_u D_v + k^2(D_u\mu_v - D_v\mu_u) + \mu_u\mu_v < 0$$

Roots have the form

$$\left\{k^2\right\}_{1,2} = \frac{D_v\mu_u - D_u\mu_v \pm \sqrt{(D_u\mu_v - D_v\mu_u)^2 - 4\mu_u\mu_v D_u D_v}}{2D_u D_v}$$

The Turing condition requires the range of k^2 values to be positive and finite. Hence, we get

$$D_v/D_u > \mu_v/\mu_u \tag{S1.2.7}$$

and

$$(D_u\mu_v - D_v\mu_u)^2 - 4\mu_u\mu_v D_u D_v > 0.$$

This inequality can be rewritten in the canonical form:

$$(\frac{\mu_v}{\mu_u} - (3-2\sqrt{2})\frac{D_v}{D_u})(\frac{\mu_v}{\mu_u} - (3+2\sqrt{2})\frac{D_v}{D_u}) > 0 \tag{S1.2.8}$$

Due to Eq (S1.2.7) the second multiplier is always less than zero. Finally, we get

$$\frac{D_v}{D_u} > \frac{\mu_v}{\mu_u}\frac{1}{3-2\sqrt{2}}. \tag{S1.2.9}$$

For rather small difference between μ_v and μ_u diffusion coefficients should differ in 6 times to supply Turing instability that is not possible for protein molecules of approximately same size.

Appendix S1.3. Turing instability of the modified system

Here we analyse the extended Gierer-Meinhardt model defined by equation:

$$\begin{cases} \dfrac{\partial u}{\partial t} = & \rho\dfrac{(b+u)^2}{v} - \mu_u u - k_1 wu + k_{-1}(w_0 - w) + D\Delta u \\ \dfrac{\partial v}{\partial t} = & \rho(b+u)^2 - \mu_v v + D\Delta v \\ \dfrac{\partial w}{\partial t} = & -k_1 wu + (k_{-1} + \mu_u)b \end{cases} \tag{S1.3.1}$$

At first, let us find the stationary points of the reaction part of the system (S1.3.1):

$$\begin{cases} 0 = & \rho\dfrac{(u+w_0-w)^2}{v} - \mu_u u - k_1 wu + k_{-1}(w_0 - w) \\ 0 = & \rho(u+w_0-w)^2 - \mu_v v \\ 0 = & -k_1 wu + (k_{-1}+\mu_u)(w_0 - w) \end{cases} \tag{S1.3.2}$$

Stationary point of the modified system In this section we show that the modified system has the unique stationary point.

$$\begin{cases} 0 = & \rho\dfrac{(u+w_0-w)^2}{v} - \mu_u u - k_1 wu + k_{-1}(w_0 - w) \\ 0 = & \rho(u+w_0-w)^2 - \mu_v v \\ 0 = & -k_1 wu + (k_{-1}+\mu_u)(w_0 - w) \end{cases} \tag{S1.3.3}$$

After the transformation:

$$\begin{cases} 0 = & \rho\dfrac{(u+w_0-w)^2}{v} - \mu_u(u+w_0-w) - k_1 wu + (k_{-1}+\mu_u)(w_0 - w) \\ 0 = & \rho(u+w_0-w)^2 - \mu_v v \\ 0 = & -k_1 wu + (k_{-1}+\mu_u)(w_0 - w) \end{cases}$$

Next substitute $u^* = (u + w_0 - w)$ and reduce the system:

$$\begin{cases} 0 = & \rho\dfrac{u^{*2}}{v} - \mu_u u^* \\ 0 = & \rho u^{*2} - \mu_v v \end{cases}$$

The stationary points satisfy Eq (4) of the main text:

$$\begin{cases} \mu_v/\mu_u = & u + w_0 - w \\ v = & \rho\mu_v/\mu_u^2. \end{cases} \tag{S1.3.4}$$

As $\overline{v}$ is found, next we find the rest two variables by joining the first equation derived now and the third equation of (S1.3.3):

$$\begin{cases} \mu_v/\mu_u = & u + w_0 - w \\ 0 = & -k_1 w u + (k_{-1} + \mu_u)(w_0 - w) \end{cases} \tag{S1.3.5}$$

After transformations:

$$\mu_u k_1 w^2 + [k_1(\mu_v - w_0\mu_u) + \mu_u(k_{-1} + \mu_u)] - w_0\mu_u(k_{-1} + \mu_u) = 0$$

Let designate $\beta_\pm = \pm k_1(\mu_u w_0 - \mu_v) + (k_{-1} + \mu_u)\mu_u; \gamma = (k_{-1} + \mu_u)\mu_v$ and the equation could be written as:

The discriminant could be written as:

$$D = \beta_+^2 + 4\gamma k_1 \mu_u > 0$$

For the final solution we get:

$$\begin{aligned} u &= \frac{\sqrt{\beta_+^2 + 4\gamma k_1\mu_u} - \beta_+}{2k_1\mu_u} \\ w &= \frac{\sqrt{\beta_+^2 + 4\gamma k_1\mu_u} - \beta_-}{2k_1\mu_u} \\ v &= \frac{\mu_v \rho}{\mu_u^2} \end{aligned} \tag{S1.3.6}$$

Let us introduce an auxiliary parameter β, γ:

$$\beta_\pm = \pm k_1(\mu_u w_0 - \mu_v) + (k_{-1} + \mu_u)\mu_u; \;\; \gamma = (k_{-1} + \mu_u)\mu_v$$

In these notations the stationary concentrations are of the form:

$$\begin{aligned} \overline{u} &= \frac{\sqrt{\beta_+^2 + 4\gamma k_1\mu_u} - \beta_+}{2k_1\mu_u} \\ \overline{w} &= \frac{\sqrt{\beta_+^2 + 4\gamma k_1\mu_u} - \beta_-}{2k_1\mu_u} \\ \overline{v} &= \frac{\mu_v \rho}{\mu_u^2} \end{aligned} \tag{S1.3.7}$$

Note, that the stationary value of v does not depend on any adsorption parameters (w_0, k_1, k_{-1}).

Reaction stability of the stationary point. Let us build the Jacobian in the stationary point:

$$\mathbb{M} = \begin{pmatrix} -k_1\overline{w} - \mu_u + 2\rho\frac{\overline{u}+w_0-\overline{w}}{\overline{v}} & -\rho\left(\frac{\overline{u}+w_0-\overline{w}}{\overline{v}}\right)^2 & -k_{-1} - k_1\overline{u} - 2\rho\frac{\overline{u}+w_0-\overline{w}}{\overline{v}} \\ 2\rho(\overline{u}+w_0-\overline{w}) & -\mu_v & -2\rho(\overline{u}+w_0-\overline{w}) \\ -k_1\overline{w} & 0 & -k_{-1} - k_1\overline{u} - \mu_u \end{pmatrix}$$

After the manipulations with rows and columns in the matrix $(\mathbb{M} - \lambda\mathbb{E})$ we get the simpler matrix with the same determinant:

$$\begin{vmatrix} -\mu_u + 2\rho\frac{\overline{u}+w_0-\overline{w}}{\overline{v}} - \lambda & -\rho\left(\frac{\overline{u}+w_0-\overline{w}}{\overline{v}}\right)^2 & 0 \\ 2\rho(\overline{u}+w_0-\overline{w}) & -\mu_v - \lambda & 0 \\ -k_1\overline{w} & 0 & -k_{-1} - k_1\overline{u} - \mu_u - k_1\overline{w} - \lambda \end{vmatrix} \quad \text{(S1.3.8)}$$

Now it become clear that $\mathbb{M}$ has three eigenvalues; at least one of them has a simple form and is always negative:

$$\lambda_1 = -(k_{-1} + \mu_u + k_1(\overline{w} + \overline{u}))$$

Also, considering Eq (S1.3.7), one can prove that

$$\overline{u} - \overline{w} + w_0 = \mu_v/\mu_u$$

Substituting this expression together with Eq (S1.3.7) into Eq (S1.3.8), we conclude that the upper left minor have the same form as the characteristic matrix of the original system (S1.2.3). Since one eigenvalue is always negative and two other eigenvalues have the same expression as eigenvalues of the classical system, we see that the reaction stability condition also looks like Eq (S1.2.4).

Reaction-diffusion saddle-type instability of the stationary point. The diffusion-augmented Jacobian has the complicated form:

$$\begin{vmatrix} -k_1\overline{w} - \mu_u + 2\rho\frac{\overline{u}+w_0-\overline{w}}{\overline{v}} - k^2D - \lambda & -\rho\left(\frac{\overline{u}+w_0-\overline{w}}{\overline{v}}\right)^2 & -k_{-1} - k_1\overline{u} - 2\rho\frac{\overline{u}+w_0-\overline{w}}{\overline{v}} \\ 2\rho(\overline{u}+w_0-\overline{w}) & -\mu_v - k^2D - \lambda & -2\rho(\overline{u}+w_0-\overline{w}) \\ -k_1\overline{w} & 0 & -k_{-1} - k_1\overline{u} - \mu_u - \lambda \end{vmatrix} = 0$$

After the same transformations as were performed in (S1.3.8) the sufficient condition can be written as the following:
The determinant

$$\begin{vmatrix} \mu_u - k^2D - \lambda & -\mu_u^2/\rho & -k^2D \\ 2\rho\mu_v/\mu_u & -\mu_v - k^2D - \lambda & 0 \\ -k_1\overline{w} & 0 & -k_{-1} - k_1(\overline{u}+\overline{w}) - \mu_u - \lambda \end{vmatrix} \quad \text{(S1.3.9)}$$

should have only one root with a positive real part for a finite positive range of k.

Formulation of this condition in an explicit analytical form is out of question. However, one can note that the upper left minor of the matrix (S1.3.9) is precisely equal to the matrix (S1.2.5) of the classical system. It may help if one wants to add an adsorption to some other two-component reaction-diffusion system or to generalize the results of the present study.

References

1. Satnoianu RA, Menzinger M, Maini PK. Turing instabilities in general systems. Journ Math Biol. 2010;41(6):493–512. doi:10.1007/s002850000056.
