# Supplementary material for: Morphogene adsorption as a Turing instability regulator: Theoretical analysis and possible applications in multicellular embryonic systems
Source: PLoS One. 2017 Feb 7;12(2):e0171212. doi: 10.1371/journal.pone.0171212 (PMC5295678; doi:10.1371/journal.pone.0171212)
Supplement: S2 Appendix — In the appendix we perform description of flux sweeping computational scheme as reproduced from ref. [25] with adaptation to our case. (PDF) [file pone.0171212.s002.pdf]

Full paper: Nesterenko A.M., Kuznetsov M.B., Korotkova D.D., Zaraisky A.G. Morphogene Adsorption on the Extacellular Matrix as a Possible Turing Instability Regulator in Multicellular Embryonic Systems. *PLoS ONE* (2017): 10.1371/journal.pone.0171212

Appendix S2. Flux sweeping algorithm

The description of flux sweeping is reproduced from the book [1] written in Russian with adaptation to our case. Let us rewrite diffusion equation

$$\frac{\partial u}{\partial t} = D\frac{\partial^2 u}{\partial x^2}$$

into the flux form:

$$\frac{\partial u}{\partial t} = \frac{\partial j}{\partial x} \qquad D\frac{\partial u}{\partial x} = j$$

Let us consider the system on the linear grid of M points, where $x_m = mh$, $h = L/M$. Discrete equations at the grid nodes are the following:

$$\begin{aligned} \frac{u_m^{n+1} - u_m^n}{\tau} &= \frac{j_{m+1/2} - j_{m-1/2}}{h}, \quad m = 1, 2, .., M-1, \\ D\frac{u_{m+1}^{n+1} - u_m^{n+1}}{h} &= j_{m+1/2}, \quad m = 0, 1, 2, .., M-1. \end{aligned} \tag{S2.1}$$

Let us write sweeping relation in the following form:

$$u_m^{n+1} = P_m\, j_{m+1/2} + R_m, \quad m = 0, 1, 2, .., M-1,$$

where P_m and R_m — supplementary ("sweeping") coefficients that should be determined. First pair of these coefficients can be determined from left edge condition $\partial u(0,t)/\partial x = 0$ (we consider Neuman condition):

$$P_0 = 2\tau/h, \quad R_0 = u_0^n. \tag{S2.2}$$

Next supplementary coefficients P_m, R_m can be determined recurrently at the first stage (forward sweeping):

$$\begin{aligned} A &= 1 + \frac{h}{\tau}\left(P_m + \frac{h}{D}\right), \\ P_{m+1} &= \frac{1}{A}\left(P_m + \frac{h}{D}\right), \\ R_{m+1} &= \frac{1}{A}\left[\left(P_m + \frac{h}{D}\right)\frac{h}{\tau}u_{m+1}^n + R_m\right]. \end{aligned} \tag{S2.3}$$

At the second stage (backward sweeping) we starts with calculating u_M^{n+1} from right edge condition $\partial u(L,t)/\partial x = 0$:

$$\begin{aligned} u_M^{n+1} &= \frac{2\tau D R_{M-1} + (h + P_{M-1}\,D)\,h u_M^n}{h^2 + hP_{M-1}D + 2\tau D}, \\ j_{M-1} &= \frac{h}{2\tau}\left(u_M^n - u_M^{n+1}\right). \end{aligned} \tag{S2.4}$$

Next, values of variable u and its flux j in the left grid points are calculated sequentially:

$$\begin{aligned} j_{m-1/2} &= j_{m+1/2} - \frac{h}{\tau} u_m^{n+1} + \frac{h}{\tau} u_m^n, \\ u_{m-1}^{n+1} &= P_{m-1}\, j_{m-1/2} + R_{m-1}. \end{aligned} \tag{S2.5}$$

Equations (S2.3),(S2.5) are called flux sweeping equations, whereas Eq (S2.2) and Eq (S2.4) can be modified according to edge conditions.

References

1. Fedorenko RP. Introduction to Computational Physics. Moscow, Russia: MPTI; 1994.
